# [Corrigendum] Silencing of Rac1 modifies lung cancer cell migration, invasion and actin cytoskeleton rearrangements and enhances chemosensitivity to antitumor drugs

**DOI:** 10.3892/ijmm.2026.5765

**Published:** 2026-02-12

**Authors:** Qing-Yong Chen, Li-Qun Xu, De-Min Jiao, Qing-Hua Yao, Yan-Yi Wang, Hui-Zhen Hu, Yu-Quan Wu, Jia Song, Jie Yan, Li-Jun Wu

Int J Mol Med 28: 769-776, 2011; DOI: 10.3892/ijmm.2011.775

Following the publication of this paper, it was drawn to the Editor's attention by an interested reader that, for the Transwell migration and invasion assay experiments shown in Fig. 3A and 3C respectively on p. 772, one and two pairs of data panels respectively were overlapping, such that data which were intended to show the results of differently performed experiments had apparently been derived from the same original sources. In addition, in Fig. 1 on p. 771, the same data panel had apparently been included to show the results of (C) strong cytoplasmic Rac1 expression and (E) weak cytoplasmic Rac1 expression in lung squamous cell carcinoma tissues.

Upon contacting the authors about these issues, they realized that certain of the data had inadvertently been included in Figs. 1 and 3 incorrectly. The revised versions of Figs. 1 and 3, now featuring the correct data for weak cytoplasmic expression in Fig. 1E and the correct data panels for the 801D-shRNA control and 801D-NSC23766 experiments in Fig. 3A and C respectively, are shown opposite and on the next page. The authors wish to emphasize that the errors made in assembling the data in this pair of figures did not affect the overall conclusions reported in the paper. The authors are grateful to the Editor of *International Journal of Molecular Medicine* for granting them this opportunity to publish a Corrigendum, and apologize to both the Editor and the readership for any inconvenience caused.

## Figures and Tables

**Figure 1:**
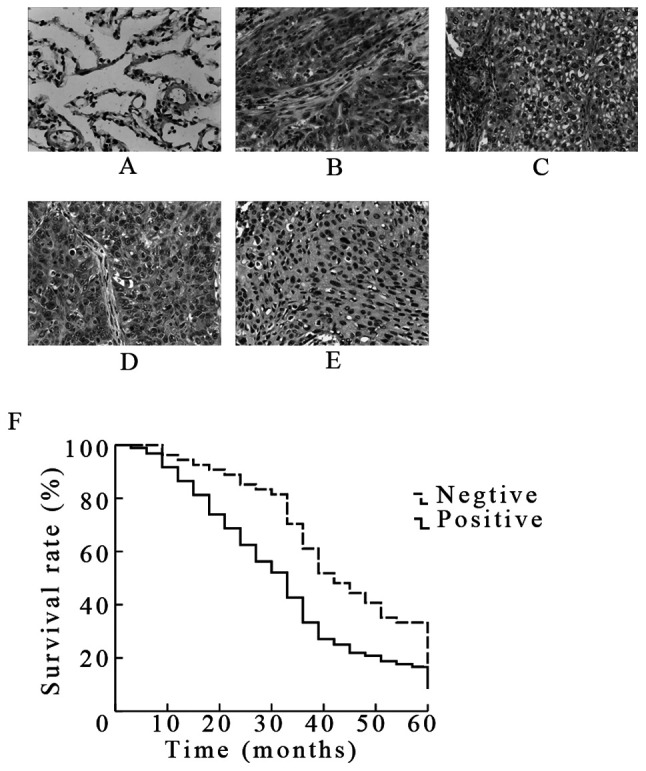
Expression of Rac1 in lung cancer tissues and its relation to overall survival. (A) Negative immunostaining for Rac1 in normal lung tissues. In contrast, Rac1 showed strong cytoplasmic staining in (B) lung adenocarcinoma and (C) lung squamous cell carcinoma (SCC), weak cytoplasmic staining in (D) lung adenocarcinoma and (E) lung SCC. (F) Kaplan-Meier survival curves were constructed, and the difference between the Rac1-positive and Rac1-negative groups was analyzed by log-rank test. Magnification, ×200.

**Figure 3:**
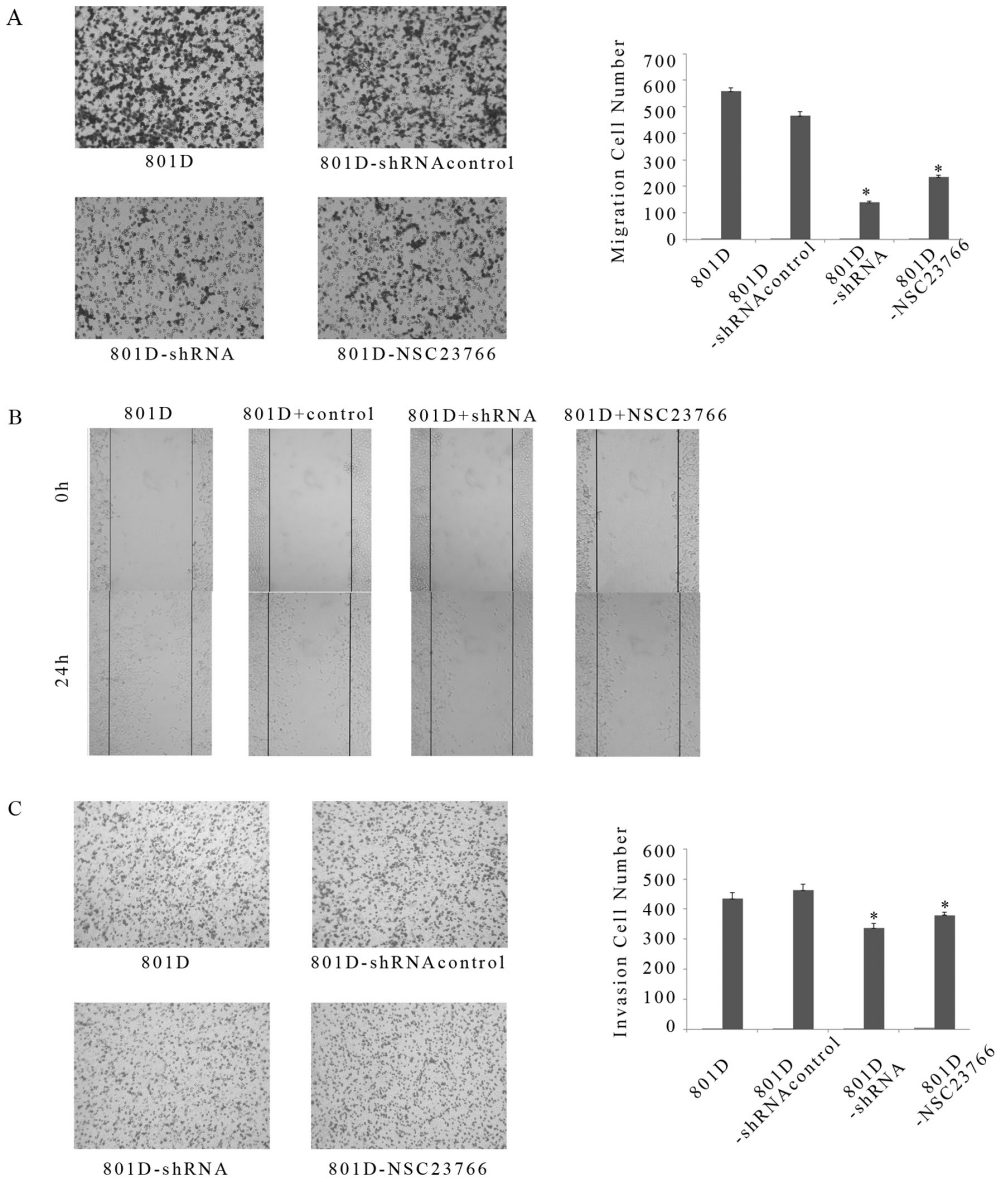
Transwell insert and wound healing assay showing that Rac1 regulates cell migration and invasion in vitro. After initial equilibrium, 801D cells un-transfected or transfected with Rac1 or negative control and treated with NSC23766 suspended in fresh medium without fetal bovine serum were added to the insert. (A) The migratory cell number of 801D transfected with Rac1 was significantly less than that of 801D cells transfected with negative control or un-transfected. (B) Confluent cell monolayers were wounded with a pipette tip. Wound closure was monitored by microscopy at the indicated times. (C) For the invasion assay, the inserts were coated with ECM and then repeated as for the migration assay. The invasive cell number of 801D transfected with Rac1 was also significantly lower than that of 801D cells transfected with negative control. Effects of NSC23766 treatment on the invasion of lung cancer cells is very similar to the effects of Rac1-shRNA. ^*^P<0.05 compared with control.

